# *Trichuris ovis*: Prevalence, Molecular Advances, Immunopathogenesis, and Diagnosis of a Neglected Ruminant Whipworm

**DOI:** 10.3390/ijms27219792

**Published:** 2026-10-31

**Authors:** Tamara Ilić, Ana Milosavljević, Oliver Stevanović, Nemanja M. Jovanović

**Affiliations:** 1 Department of Parasitology and Parasitic Diseases, Faculty of Veterinary Medicine, University of Belgrade, Bulevar oslobođenja 18, 11000 Belgrade, Serbia; tamara@vet.bg.ac.rs (T.I.); ana.milosavljevic@vet.bg.ac.rs (A.M.); 2 PI Veterinary Institute of the Republic of Srpska “Dr. Vaso Butozan”, 78000 Banja Luka, Bosnia and Herzegovina; oliver.stevanovic@virs-vb.com

**Keywords:** *Trichuris ovis*, whipworm, ruminants, molecular systematics, immunopathogenesis, coprological diagnosis, mitochondrial markers

## Abstract

*Trichuris ovis* is a widespread whipworm of the cecum and colon of domestic ruminants, transmitted through resistant eggs that persist on pastures for years. Although most infections are subclinical, heavy infections in young animals cause typhlitis and colitis. This narrative review summarizes current knowledge on *T. ovis*, emphasizing molecular systematics, immunopathogenesis, and diagnosis. Complete mitochondrial genomes have confirmed *T. ovis* as distinct from *T. discolor* and *T. skrjabini*, but discordance between ribosomal and mitochondrial markers leaves its relationship with *T. globulosa* unresolved and suggests cryptic lineages. Reported prevalence ranges from below 1% to over 70% and depends on the diagnostic method, as coprological examination cannot distinguish *Trichuris* species. Immunological knowledge derives mainly from the *T. muris* model, in which T helper 2 (Th2) responses mediate worm expulsion and excretory–secretory proteins modulate host immunity, whereas data for ruminants remain scarce. Flotation solutions of higher specific gravity improve egg detection, and combined nuclear and mitochondrial markers are required for reliable species identification. Control should rely on integrated parasite management that prioritizes reducing environmental contamination. Remaining gaps include anthelmintic efficacy and resistance in *T. ovis* and molecular data from underrepresented regions, including the Balkans.

## 1. Introduction

The genus *Trichuris* Roederer, 1761 (family Trichuridae) comprises a large number of nematode species that parasitize the cecum and large intestine of mammals, including domestic and wild ruminants, pigs, carnivores, rodents, primates, and humans. More than 70 species of *Trichuris* have been described, and most of them are considered host-specific. Members of the genus *Trichuris* have a cosmopolitan distribution and are classified as geohelminths: the life cycle is direct, without an intermediate host, and the eggs embryonate in the environment, where the infective larva develops within them. Their particular epidemiological importance stems from the high resistance of the eggs to adverse environmental conditions and their ability to persist in soil for long periods, which allows infection to be maintained over time in populations of susceptible animals. This problem is especially pronounced in extensive management systems, where continuous pasture contamination favors the accumulation of infective eggs and the reinfection of hosts [1,2].

The most important *Trichuris* species in domestic ruminants are *Trichuris ovis*, *T. discolor*, *T. skrjabini*, and *T. globulosa*. These species share certain biological features but differ in host range, morphometric characteristics, and geographic distribution. *Trichuris ovis* is the species most frequently reported in sheep and goats, but it has also been confirmed in cattle and wild ruminants [3,4]. *Trichuris discolor* parasitizes mainly cattle, *T. skrjabini* parasitizes small ruminants and camels, and *T. globulosa* parasitizes mainly camels [5]. Reliable differentiation of these species on the basis of morphology alone is difficult, which has direct consequences for the interpretation of epidemiological data and for diagnosis.

Although *T. ovis* has traditionally been regarded as a parasite of relatively low pathogenicity, high-intensity infections, particularly in lambs, kids, and calves, can cause severe damage to the cecal and colonic mucosa [4]. The thin anterior part of the parasite’s body penetrates the mucosal epithelium, leading to local inflammation, hyperemia, and hemorrhage. In heavy infections, diffuse inflammation of the cecal and colonic mucosa develops, accompanied by impaired mucosal function and clinically manifest disease [4]. Low-intensity infections, which predominate under natural conditions, are usually asymptomatic or accompanied by nonspecific clinical signs. Because the impact of these subclinical and chronic infections on weight gain and production parameters has not yet been quantified, the actual health and economic importance of this species is probably underestimated [1,6,7].

In addition to its veterinary and production importance, the genus *Trichuris* has in recent years been a focus of molecular systematics. Molecular analyses have shown that morphologically defined species often encompass several genetically distinct lineages. Analyses of complete mitochondrial genomes have indicated the existence of multiple *Trichuris* species in humans, nonhuman primates, and pigs from different geographic regions [8], and in humans the species *T. incognita*, which conventional diagnostic methods do not distinguish from *T. trichiura*, has also been confirmed [9]. In ruminants, sequencing of the complete mitochondrial genomes of *T. ovis* and *T. discolor* [10] and of *T. skrjabini* [11] confirmed the status of these taxa as separate species and provided new genetic markers for diagnosis, population genetics, and molecular epidemiology [12,13,14]. At the same time, the discordance between ribosomal and mitochondrial markers in delimiting *T. ovis* and *T. globulosa* [5,15] shows that the taxonomic status of *T. ovis* has still not been fully resolved.

Existing reviews of *T. ovis* are brief or regionally limited [4,16] and do not systematically cover the molecular taxonomy, immunopathogenesis, and molecular diagnostic methods for this species. The aim of this review is therefore to integrate and critically evaluate, on the basis of the available literature, current knowledge on *T. ovis* in domestic ruminants, with an emphasis on molecular systematics, immunopathogenesis, and diagnosis, together with an overview of the biology of the parasite, the epidemiological patterns of infection, and control measures. Relevant publications were identified by searching PubMed, Scopus, Web of Science, Google Scholar, and CAB Abstracts, without a lower date limit and up to October 2026, using the terms “*Trichuris ovis*”, “*Trichuris*” combined with “sheep”, “goat”, “cattle”, or “ruminant”, “trichuriasis”, “trichuriosis” and “whipworm”. Peer-reviewed articles, books, theses, conference proceedings, and patents were considered, including selected publications in languages other than English that provide historical or regional data. Sources were selected according to their relevance to the taxonomy, biology, epidemiology, pathogenesis, immunology, diagnosis, and control of *T. ovis* and other *Trichuris* species of ruminants. Where data on *T. ovis* were lacking, studies on model species (*T. muris*, *T. suis*, and *T. trichiura*) were included and are identified as such in the text. The reference lists of the retrieved publications were screened for additional sources.

## 2. Taxonomy and Molecular Systematics of *Trichuris ovis*

The species was originally described as *Trichocephalus ovis* Abildgaard, 1795, which represents the original combination. The current name, *Trichuris ovis*, results from a nomenclatural decision at the genus level, by which the name *Trichuris* Roederer, 1761 was given priority over *Trichocephalus* Goeze, 1782.

During the 19th and early 20th centuries, the identification of *Trichuris* species in ruminants relied exclusively on the morphological characteristics of adults, which led to inconsistent nomenclature and misidentifications. Records from Iceland provide an illustrative example: Einarsson [17] reported the nematode *Trichocephalus affinis*, whereas Dungal and Gíslason [18] reported a high prevalence (52%) of *Trichocephalus dispar* in sheep in southwestern Iceland, although this name refers to a species that parasitizes humans (now *T. trichiura*). According to Richter [19], both records most probably refer to *T. ovis*.

Particular attention in morphological identification was paid to the structure of the male, especially the spicule length, the shape and dimensions of the spicular sheath, and the arrangement of spines on its surface, although individual authors assessed the diagnostic value of these characteristics differently. A specific taxonomic problem was the differentiation of *T. ovis* from *T. globulosa*. Sprehn [20], and subsequently Baylis [21] and Chandler [22], emphasized the importance of the spicular sheath, spicule length, and spine arrangement for differentiating these two species [23,24]. Later studies, however, showed that some morphometric characteristics, particularly spicule length, may overlap between *T. ovis* and *T. globulosa*, which called into question their reliability as stand-alone taxonomic criteria.

In the late 20th century, biochemical (isoenzyme) and molecular methods were introduced into the study of taxonomic relationships within the genus *Trichuris*. Cutillas et al. [23] conducted a comparative morphological, biometric, and genetic study of *T. ovis* and *T. globulosa* and concluded that the two forms could be regarded as a single species, treating *T. globulosa* as a synonym of *T. ovis*. This view was supported by Oliveros et al. [25], who found no differences in the sequences of the second internal transcribed spacer of ribosomal DNA (ITS2) between isolates identified as *T. ovis* and *T. globulosa*. Oliveros and Cutillas [24] subsequently redescribed *T. ovis* on the basis of morphological, biometric, isoenzyme, and molecular data and accepted *T. globulosa* as its synonym.

Molecular markers have also enabled reliable delimitation of *T. ovis* from other species in ruminants. On the basis of first internal transcribed spacer (ITS1) and ITS2 sequences and the partial mitochondrial 16S gene, Callejón et al. [3] clearly separated *T. ovis* from *T. discolor* from cattle, while also pointing to possible cryptic species within *T. discolor* (populations from Spain and Iran). The status of these two species was confirmed by Liu et al. [10] through analysis of their complete mitochondrial genomes (13,946 bp in *T. ovis* and 13,904 bp in *T. discolor*), each of which contains 37 genes arranged in the same order as in *T. trichiura* and *T. suis*. Analysis of the complete mitochondrial genome (14,011 bp) also confirmed the status of *T. skrjabini* as a separate species [11]. Analyzing partial sequences of the cytochrome c oxidase subunit 1 (*cox1*), NADH dehydrogenase subunit 5 (*nad5*), and cytochrome b (*cytb*) genes in *T. ovis* isolates from different hosts, Wang et al. [26] found intraspecific differences of 0–1.9%, compared with 24.3–56.4% between *Trichuris* species, confirming the usefulness of these markers for species delimitation and molecular epidemiology.

The development of molecular phylogenetics, however, led to a reappraisal of the synonymy of *T. ovis* and *T. globulosa*. Callejón et al. [27] analyzed nuclear (18S rRNA, triosephosphate isomerase [*TPI*]) and mitochondrial (*cox1*, *cob*) genes of the genus *Trichuris* and showed that the highest phylogenetic resolution is achieved by the combined analysis of multiple markers. In a study of *T. globulosa* from camels in Iran and of *Trichuris* species from sheep in South Africa, Callejón et al. [5] found no significant differences in ITS2 sequences between *T. ovis* and *T. globulosa*, but partial sequences of the mitochondrial *cox1* and *cytb* genes indicated that *T. globulosa* represents a separate genetic lineage. The same authors also identified a distinct genetic lineage of *T. ovis* from sheep in South Africa, closely related to *T. globulosa* populations from camels. In camels from Kuwait, Henedi et al. [15] distinguished two groups on the basis of the *cox1*, 12S rRNA, and 16S rRNA genes, one related to *T. globulosa* and the other to *T. ovis*, whereas the ITS2 region lacked sufficient resolution to separate them. The shape of the spicular sheath did not agree with the molecular findings, leading the authors to conclude that these parasites probably represent a *T. globulosa* species complex and that this morphological characteristic is of limited diagnostic value.

Taken together, these studies indicate that morphological characteristics, primarily the spicular sheath and spicule length, retain some diagnostic value but show considerable variability and overlap between species [5,15]. The ribosomal ITS2 region reliably separates species that parasitize different hosts but lacks sufficient resolution to distinguish *T. ovis* from *T. globulosa*, for which mitochondrial markers (*cox1*, *cob*/*cytb*, 12S and 16S rRNA) are more suitable [3,5,15]. Therefore, combining morphological and morphometric data with sequences of ribosomal and mitochondrial markers is recommended for the reliable identification of *T. ovis*. Recent phylogenetic analyses also indicate that some traditionally defined species, including *T. ovis*, contain distinct genetic lineages or potential species complexes whose taxonomic status remains to be clarified through an integrative approach.

## 3. Biology of the Parasite

### 3.1. Life Cycle

**Figure 1:**
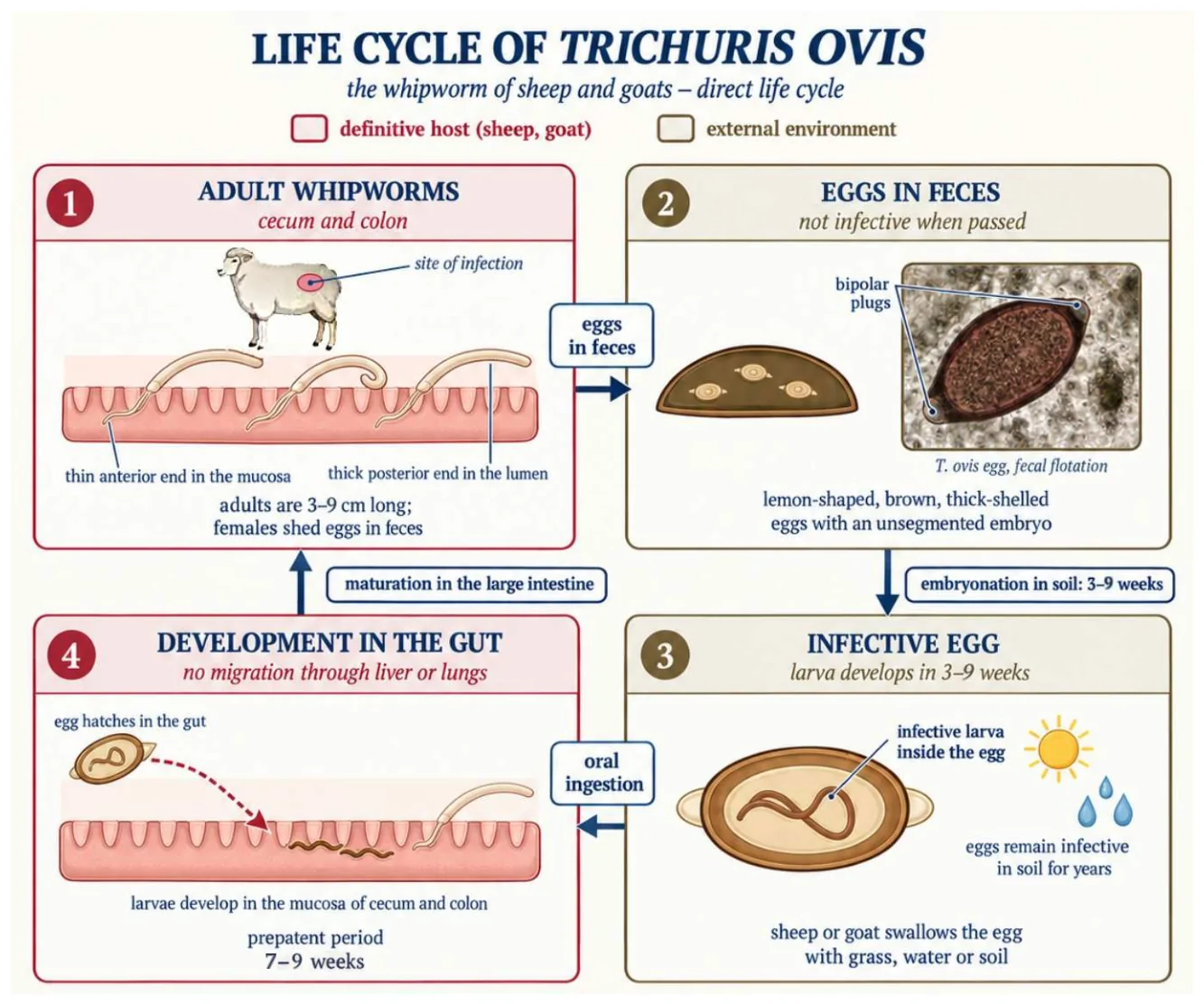
Life cycle of *Trichuris ovis*.

The life cycle of *T. ovis* is direct and proceeds without an intermediate host [28] (Figure 1). Adult females lay unembryonated eggs, which are shed into the environment with the feces. Under suitable conditions of temperature and humidity, the first-stage larva (L1), which is the infective form of the parasite, develops within the egg. Under optimal conditions, embryonation takes approximately three weeks, whereas at lower temperatures (6–20 °C) it may take 1–2 months. Embryonated eggs are highly resistant to adverse environmental conditions and can remain infective for several years, thus representing a long-lasting source of infection on pastures [1,2].

Ruminants become infected by ingesting embryonated eggs with contaminated feed or water. In the intestine, the polar plugs are degraded and the L1 hatch and penetrate the glands of the distal ileum and cecum [2,6]. According to older reports, the larva first resides for 2–10 days in the glands of the anterior small intestine and then migrates to the cecum [1,29].

All four molts take place in the epithelium of the cecal and proximal colonic mucosa, within a syncytial tunnel that the larva forms in the crypt epithelium [30]. Upon completion of development, the extremely thin anterior part of the adult worm remains embedded in the epithelial tunnel, while the posterior part protrudes freely into the intestinal lumen. This position allows the parasite to attach to host tissue, feed, copulate, and continuously release eggs at the same time [6,30]. The prepatent period is approximately 7–9 weeks [1].

### 3.2. Molecular Mechanisms of Larval Hatching

Larval hatching is the first step of infection and, according to findings in the model species *T. muris*, depends on the intestinal microbiota of the host. Contact of bacteria with the polar plugs leads to their asymmetric degradation before the larva emerges, and the results indicate that a chitinase released by the larva from inside the egg participates in plug degradation [31]. Goulding et al. [32] showed that protease inhibitors block bacteria-induced hatching and, using transcriptomic analysis of *T. muris* eggs during embryonation, found increased expression of serine proteases (family S01A) in fully embryonated eggs. Inhibition of serine proteases completely prevented hatching, from which it was concluded that serine proteases of both bacterial and larval origin are involved in this process.

Hatching mechanisms are not the same in all *Trichuris* species. Bacteria that induce hatching of *T. muris* eggs did not induce hatching of *T. suis* eggs, indicating that individual species are adapted to the microbiota of their hosts [33]. Findings from model species therefore cannot be directly applied to *T. ovis*, for which the hatching conditions, the role of the ruminant microbiota, and the molecular mechanisms of epithelial invasion have not yet been investigated.

The direct life cycle, the high resistance of embryonated eggs, and long-term pasture contamination are the key biological features that enable *T. ovis* to persist in ruminant herds and provide the basis for interpreting the epidemiological patterns described in the next section.

## 4. Epidemiological Patterns of *Trichuris ovis* Infection

*Trichuris ovis* occurs on all continents where sheep and goats are raised, and the frequency of infection depends on climatic conditions, management system, stocking density, host age, and the use of antiparasitic measures [2,34]. When prevalence data are interpreted, the diagnostic method used must be taken into account. Coprological examination cannot reliably distinguish *T. ovis* eggs from those of other *Trichuris* species in ruminants, so such findings refer to *Trichuris* spp. Species identification is possible on the basis of adult morphology after necropsy or slaughter, but this approach also has limitations (Section 2), whereas molecular methods allow reliable species determination [12]. The importance of this problem is illustrated by a study in Xinjiang (China), in which *Trichuris* spp. were detected in 86.1% of 1216 slaughtered sheep, but *T. gazellae* and *T. lani*, rather than *T. ovis*, were morphologically identified as the dominant species [35].

### 4.1. Global Distribution and Prevalence

**Figure 2:**
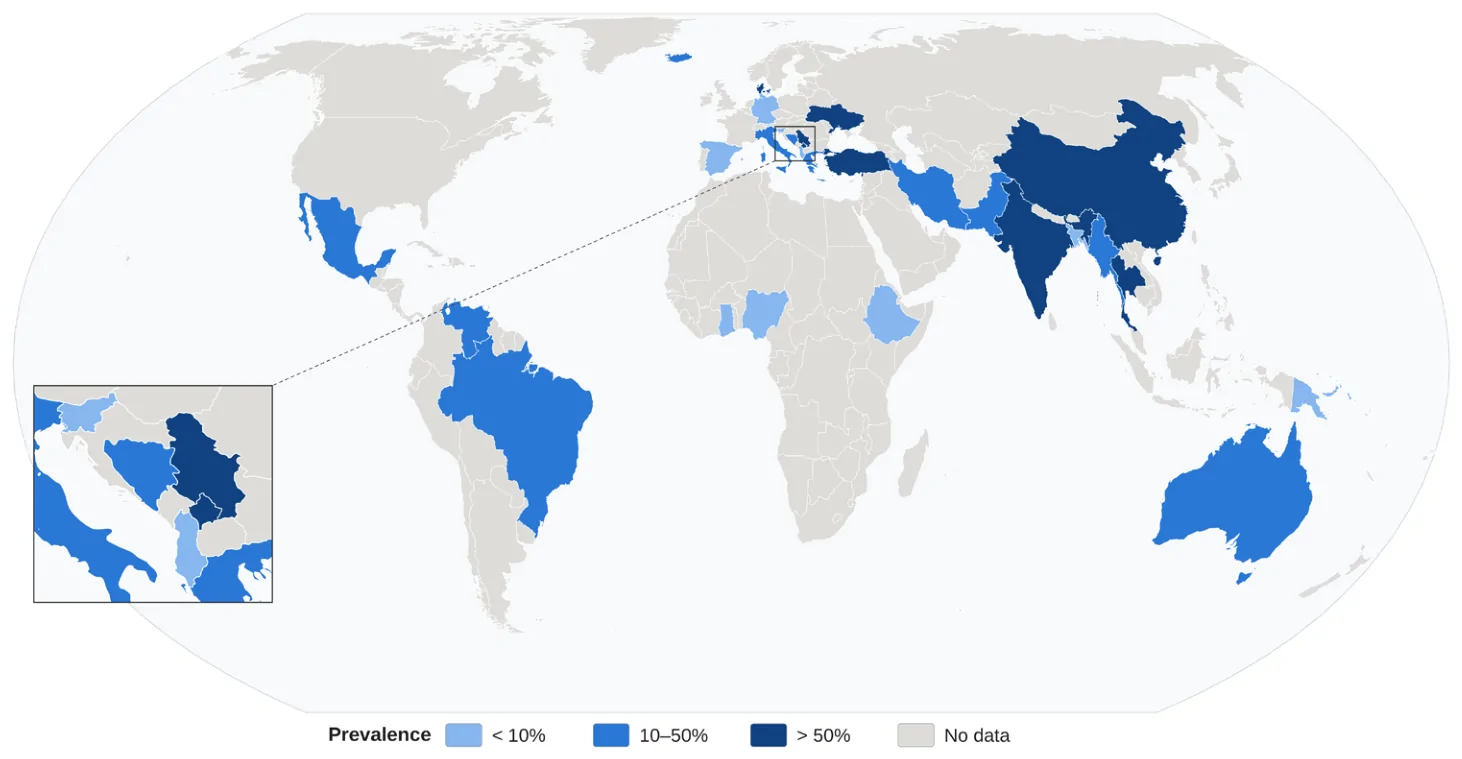
Global prevalence of *Trichuris* spp.

A review of published studies reveals heterogeneous results. Studies differ in diagnostic method (coprological examination or examination of the intestine after necropsy or slaughter), in the unit of observation (individual animal or farm/flock), in the age of the animals, and in the management system (Figure 2). The highest values generally come from studies based on intestinal examination and from extensive management systems, and the lowest from coprological studies in cattle.

Europe. The highest values in Europe were obtained by intestinal examination. In abattoirs in the Burdur region of Turkey, *T. ovis* was found in 72.0% of sheep [36]. Yevstafieva et al. [37] confirmed trichuriosis by morphological identification of adults in 65% of sheep in central and southeastern Ukraine. In Iceland, the prevalence in sheep was 35.1% [19]. In goats, high values were recorded in Denmark (33.7–63.0%) [38]. At the farm level, *Trichuris* spp. eggs were found on 48.89% of sheep farms and 42.22% of goat farms in southern Italy [39] and in 20.0% of dairy sheep flocks in Greece [40]. Other studies in Europe report low values. *Trichuris ovis* was found in 6.1% of sheep in Greece [41], in 1.6% of sheep in Albania [42], and in 0.65–6.16% of sheep and 2.56% of goats in southern Spain [43]. In the southwestern Iberian Peninsula, the prevalence in sheep was 3.2% [44]. In Germany, *Trichuris* spp. were found in 3.4% of lambs [45], and in beef cattle imported from France into Italy, in 4.1% of animals [46].

Asia. High values have been recorded in Central and South Asia. In small ruminants slaughtered in Srinagar (Kashmir, India), *Trichuris* spp. were detected in 40.46% of sheep and 50.51% of goats [47]. In Pakistan, the prevalence was 40.00% in sheep and 35.48% in goats in the Rawalpindi and Islamabad area [48] and 17.39% in sheep in Khyber Pakhtunkhwa [49]. In central Myanmar, 38.9% of sheep and 24% of goats were infected [50]. In northeastern Thailand, *Trichuris* spp. were found in 73.8% of goat herds [51]; this value refers to herds rather than to individual animals. A meta-analysis of data from Iran yielded a pooled *Trichuris* spp. prevalence of 18.42% in small ruminants [52]. Individual studies in Iran report lower values: 5.58% in sheep in Mazandaran Province [53] and 1.1% in cattle, 6.0% in sheep, and 13.0% in goats in southeastern Iran [54]. In India, a prevalence of 12.1% was recorded in sheep and goats in Jammu Province [55]. In the same region, Mir and Chishti [56] found a prevalence of 14.43% in goats. The lowest values in Asia come from coprological studies. In China, *Trichuris* spp. were detected in 0.6% of sheep and 0.4% of goats in three provinces [57], and in Bangladesh in 1.9% of sheep [58]. These data contrast sharply with the prevalence of 86.1% in sheep slaughtered in Xinjiang [35], which again highlights the influence of the diagnostic method on the results.

Africa. In the Dawuro Zone (Ethiopia), a prevalence of only 1.8% was recorded [59]. Coprological studies in West Africa also report low values. In Nigeria, *Trichuris* spp. were found in 5.9% of sheep and 4.1% of goats [60], and in Ghana in 3.3% of cattle, sheep, and goats [61].

The Americas and Oceania. A systematic review of studies from Mexico reported a *Trichuris* spp. prevalence of 2.4–12.8% in cattle, 0.4–25.1% in sheep, and 4.6% in goats [62]. In an arid zone of Venezuela, necropsy of sheep revealed a prevalence of 13.3–26.2% [63]. In Brazil, Sousa et al. [64] found a prevalence of 27.42% in sheep by intestinal examination, compared with 6.25% by fecal examination. In Papua New Guinea, *Trichuris* spp. were found in 1.8% of sheep and 3.6% of goats [65], and in a national survey of dairy goats in Australia, in 13.0% of animals [66].

Coprological versus intestinal examination. In studies that used both approaches, intestinal examination yielded higher values than coprological examination. Nwosu et al. [60] recorded a morphologically determined prevalence of 8.9% in sheep and 6.7% in goats, compared with 5.9% and 4.1% by fecal examination, and the difference was even more pronounced in the study by Sousa et al. [64] (27.42% vs. 6.25%).

Serbia and the region. In sheep in Serbia, the prevalence of *T. ovis* was 29.58% in the Zlatibor and Nišava districts in 2000–2004 [67] and 9.05% across the entire territory of Serbia in 1990–2010 [68]. In sheep kept in a pasture-based system, a prevalence of 4.94% was determined by coprological methods [69]. Considerably higher values were recorded in autochthonous goat breeds under extensive management: 37.50–57.14% on Balkan goat farms and 71.43% on Serbian White goat farms [70]. For cattle in Serbia, there is only one report, which refers to *T. discolor*, with a prevalence of 2.17% in western Serbia [71]. In neighboring countries, the prevalence in sheep in the hilly and mountainous areas of Bosnia and Herzegovina was 24.9% [72], and in Slovenia, 0.84% of cattle were found to be infected [73]. Data from the region show the same pattern as the global overview: high values in small ruminants under extensive management and low values in cattle.

### 4.2. Risk Factors

Host age and species. The most intense infections occur in very young lambs, whereas naturally acquired infections in sheep, goats, and cattle are usually not intense enough to cause clinical disease. Sheep older than eight months develop age-related resistance, and resistance to reinfection develops two to three weeks after primary infection [1,6]. Consistent with this, Gul and Tak [47] found a higher prevalence in young animals (53.8%) than in adults (32.9%), with a significantly higher prevalence in goats than in sheep. The high prevalence in adult goats of autochthonous breeds in Serbia [70] may indicate that resistance develops less effectively in goats than in sheep, but this hypothesis remains to be tested.

Season and climate. Egg embryonation requires adequate temperature and humidity, but because embryonated eggs survive for long periods, the relationship between climate and prevalence is not straightforward. In Kashmir, the prevalence in sheep increased during autumn (42.02%), peaked in winter (59.37%), declined in spring (53.22%), and was lowest in summer (30.6%); it was also higher in the dry season (55.5%) than in the rainy season (36.36%) [47]. Host sex had no significant effect on prevalence.

Management system and control measures. Infections occur most often in grazing animals, particularly in extensive systems without planned deworming and adequate hygiene measures [2]. Das et al. [74] associated a higher frequency of infection with poor hygiene and management. In southern Italy, only 24.4% of sheep farmers and 11.3% of goat farmers performed coprological examination before treatment [39], indicating that deworming is often carried out without knowledge of the actual parasitological status. The high resistance of the eggs in the environment explains why control cannot rely solely on the use of anthelmintics.

### 4.3. Host Range and Contact Between Wild and Domestic Ruminants

In addition to sheep and goats, *T. ovis* has been confirmed in cattle [3] and wild ruminants. In the Czech Republic, Vejl et al. [12] molecularly confirmed both *T. ovis* and *T. discolor* in sheep and roe deer, with *T. ovis* predominating in sheep and *T. discolor* in roe deer, which indicates an exchange of parasites between domestic and wild ruminants in shared habitats. In western Kazakhstan, in a study of helminths of sheep and saiga antelope (*Saiga tatarica*) on shared pastures, *T. ovis* was identified molecularly on the basis of the ITS1 region [75]. The prevalence of *Trichuris* spp. in deer is generally low, and the role of wild ruminants as a source of infection for domestic ruminants remains a matter of debate [76].

Findings from hosts other than ruminants suggest that the host range may be broader than previously thought. In macaques in northeastern Thailand, two *Trichuris* spp. egg samples analyzed on the basis of the 18S rRNA gene clustered with *T. ovis* from a goat, but the authors considered this finding insufficiently robust and recommended the analysis of additional markers [14].

### 4.4. Molecular Epidemiology and Population Genetics

Molecular data on *T. ovis* come from a small number of regions. Wang et al. [26] found intraspecific differences of 0–0.8% for *cox1* and *nad5* and of 0–1.9% for *cytb* among *T. ovis* isolates from different hosts in Guangdong Province (China), with all isolates clustering together. In goats from Hunan Province, the intraspecific variability of the *cox1* gene was 0–1%, compared with 23.3–27.2% between species [13]. In the same population, intraspecific variability was 0–2.7% for ITS1 and 0–4.1% for ITS2, compared with more than 45.7% and 51.3%, respectively, between *T. ovis* and other species of the genus [77].

In contrast to the genetically homogeneous *T. discolor*, high haplotype diversity of the ITS1-5.8S-ITS2 region was found in *T. ovis* from the Czech Republic [12]. Callejón et al. [5] identified a distinct genetic lineage of *T. ovis* in sheep from South Africa, closely related to *T. globulosa* populations from camels. These findings point to a genetic structure within the species that has not yet been systematically investigated. Molecular data from the Balkans are lacking, and the inclusion of molecular species confirmation in future epidemiological studies would considerably improve our understanding of the distribution and genetic diversity of *T. ovis*.

## 5. Pathogenesis and Immunity

### 5.1. Pathogenesis and Pathological Changes

Pathological changes in *T. ovis* infection are largely confined to the cecum and proximal colon, where the adult parasites embed the extremely thin anterior part of their body in the mucosal epithelium, while the posterior part remains free in the intestinal lumen [78]. This localization allows the parasite to survive and reproduce for long periods but also underlies local mucosal damage. The severity of the changes depends primarily on the number of parasites and on the age and general health of the host. In low-intensity infections, the changes are localized and mild, whereas in high-intensity infections diphtheritic typhlitis, hemorrhagic colitis, ulceration, and mucosal necrosis may develop, accompanied by diarrhea, dehydration, anemia, and growth retardation and, in the most severe cases, by death of the animals [4].

In goats naturally infected with *T. ovis*, histopathological examination showed that the embedded anterior part of the parasite was surrounded by fibroblasts and leukocytes, with mild mononuclear cell infiltration, an increased number of goblet cells, epithelial changes, and disrupted mucosal architecture around the site of penetration, without signs of fibrosis or necrosis [79]. Active hyperemia, hemorrhages, and marked lymphocytic infiltration of the mucosa have also been described in goats [1,6]. Long-term infection leads to progressive damage to the surface epithelium at the sites where the parasite is located [1], and persistent irritation of the mucosa leads to an increase in the number and size of goblet cells and to the development of catarrhal inflammation [79].

In sheep, Satish et al. [80] examined 125 cecal and colonic samples and found *T. ovis* in five of them. Grossly, hyperemia, thickening, and edema of the cecal mucosa were observed, together with raised nodules more than 5 mm in diameter. Histopathologically, the mucosa was thickened, with numerous eosinophils, an increased number of goblet cells, hyperplasia of the glandular epithelium, and marked mononuclear infiltration. Iliev et al. [7] described an infection in a flock of 12 lambs in southern Bulgaria in which diarrhea and weight loss were observed; one lamb died despite antibiotic therapy.

Infection with *T. ovis* also alters the cecal microbiota. Analysis of the 16S rRNA gene in Kashmir Merino sheep showed that the microbial communities of infected and uninfected ceca differed significantly: the infected cecum had greater diversity and higher proportions of the genera *Bacteroides*, *Prevotella*, and *Treponema*, whereas Firmicutes and Verrucomicrobiota predominated in the uninfected cecum. The microbiome of the parasite itself was dominated by the genus *Escherichia*/*Shigella*. The authors concluded that infection introduces potentially pathogenic bacteria and reduces the proportion of beneficial ones [81]. In an experimental model of trichuriosis, bacterial invasion through the damaged mucosa becomes pronounced from the development of the third larval stage (day 17) onward and intensifies with the development of the fourth larval stage and adults [82]. In pigs infected with *T. suis*, mucosal damage has been reported to favor secondary bacterial infections [83], whereas this process has not yet been investigated for *T. ovis*.

### 5.2. Immune Response: Insights from the T. muris Model

The immune response to *Trichuris* species has been studied best in the *T. muris* model in mice and, to some extent, in the *T. suis* model in pigs [30,83,84,85,86]. In resistant mouse strains, infection induces a T helper 2 (Th2) response (IL-4, IL-5, IL-13) that leads to worm expulsion, whereas a T helper 1 (Th1) response (IL-12, IL-18, IFN-γ) in susceptible strains results in chronic infection [87]. Beyond this classical dichotomy, innate lymphoid cells, basophils, dendritic cells, and regulatory T cells also participate in the response [88]. The key effector cytokine is IL-13: its neutralization prevents worm expulsion, and administration of recombinant IL-13 to susceptible mice reduced worm burdens by 87.85% [89]. The alarmin IL-33 drives the response toward the Th2 type and can induce worm expulsion even in susceptible mice [90].

Th2 cytokines alter the intestinal environment in several ways: they accelerate epithelial turnover, promote goblet cell differentiation, and alter epithelial permeability and smooth muscle contractility [87]. Cliffe et al. [91] showed that accelerated epithelial turnover in the large intestine acts as an “epithelial escalator” that expels the parasite from its niche and that this process is controlled by IL-13 and the chemokine CXCL10. IL-13 also maintains mucin sulfation in crypt goblet cells, which makes the mucus more resistant to degradation by the excretory–secretory (ES) products of the parasite; mice with reduced mucin sulfation remain susceptible to chronic infection despite a strong Th2 response [92].

The model of repeated low-dose (trickle) infections is particularly relevant for understanding natural infections in ruminants. In mice, this infection regimen leads to the slow acquisition of immunity, with a gradual shift in the CD4^+^ T cell response from the Th1 to the Th2 type, goblet cell hyperplasia, increased production of the mucin Muc5ac, and accelerated epithelial turnover; depletion of CD4^+^ T cells abrogates the acquired resistance [93]. This pattern could explain the gradual development of age-related resistance in sheep, but this has not been investigated for *T. ovis*.

### 5.3. Immunomodulation and Immune Evasion

The long-term persistence of *Trichuris* infections despite an intact host immune system is attributed to immunomodulatory molecules in the ES products of the parasite [94,95]. The best studied of these is the protein p43, which accounts for about 95% of the proteins secreted by adult *T. muris*. This protein is non-immunogenic during infection, binds to matrix proteoglycans, and binds IL-13, thereby inhibiting its function in vitro and in vivo [94]. Protein p43 also binds fatty acids and retinol; its ortholog from *T. trichiura* has similar activity, and proteins of this type are known only from nematodes of the subclass Dorylaimia [96]. The existence and function of p43 orthologs in *T. ovis* have not been investigated.

*Trichuris muris* secretes 148 identified proteins as well as extracellular vesicles containing more than 350 proteins and 56 microRNAs, many of which are associated with genes that regulate host inflammation; the vesicles are internalized by colonic organoid cells [97]. *Trichuris suis* ES proteins reduce the secretion of the proinflammatory cytokines TNF-α and IL-12p70 by macrophages and dendritic cells and promote the secretion of IL-10; this effect is reproduced by the recombinant enzymes triosephosphate isomerase and nucleoside diphosphate kinase [98]. The genomes of *T. muris*, *T. trichiura*, and *T. suis* encode numerous proteins with whey acidic protein (WAP) and cysteine-rich secretory protein, antigen 5, and pathogenesis-related 1 protein (CAP) domains, which are thought to play a role in host–parasite interactions and are being considered as vaccine candidates [99].

### 5.4. Immune Response of Ruminants to T. ovis and Open Questions

Data on the immune response of ruminants to *T. ovis* are very limited. In the reported case of infection in lambs, immunohistochemistry revealed increased numbers of CD68^+^ macrophages and CD4^+^ lymphocytes in the cecal mucosa, indicating activation of the cellular immune response, whereas granzyme B-positive cells were not found [7]. The absence of these cells suggests that cytotoxic mechanisms (natural killer [NK] cells and cytotoxic T lymphocytes) probably do not play a major role in local defense, but this conclusion, which is based on a single flock, needs to be confirmed in a larger number of animals. The marked eosinophilic infiltration and goblet cell hyperplasia observed in sheep [80] are consistent with the Th2-type response described for *T. muris*.

## 6. Diagnosis

Diagnosis of *T. ovis* infection is based on clinical suspicion, coprological examination, post-mortem findings with morphological identification of adults, and molecular methods. Each level provides different information: coprological examination confirms trichuriosis but not the species of the parasite, whereas adult morphology and molecular methods can provide reliable species identification.

### 6.1. Clinical Suspicion

Infection may be suspected on the basis of the history, epidemiological data, and clinical signs. Most natural infections are of low intensity and are asymptomatic or accompanied by mild, nonspecific signs, so they are detected only by coprological examination or at necropsy [7]. Isolated outbreaks with more severe clinical disease have also been described [78,100]. In high-intensity infections, particularly in lambs, hemorrhagic colitis and diphtheritic typhlitis develop, accompanied by diarrhea, severe anemia, dehydration, icterus, and emaciation, and deaths may occur [2,101]. Thickening of the cecal wall reduces water absorption in the large intestine, which contributes to the development of diarrhea [34].

The relationship between infection intensity and clinical signs is not always direct. In a flock of 12 lambs in which *Trichuris* eggs were found in all animals, diarrhea was observed in only two [7]. Because the clinical presentation is nonspecific, laboratory confirmation and the exclusion of other causes of diarrhea in young ruminants are necessary to establish a diagnosis.

### 6.2. Coprological Diagnosis

**Figure 3:**
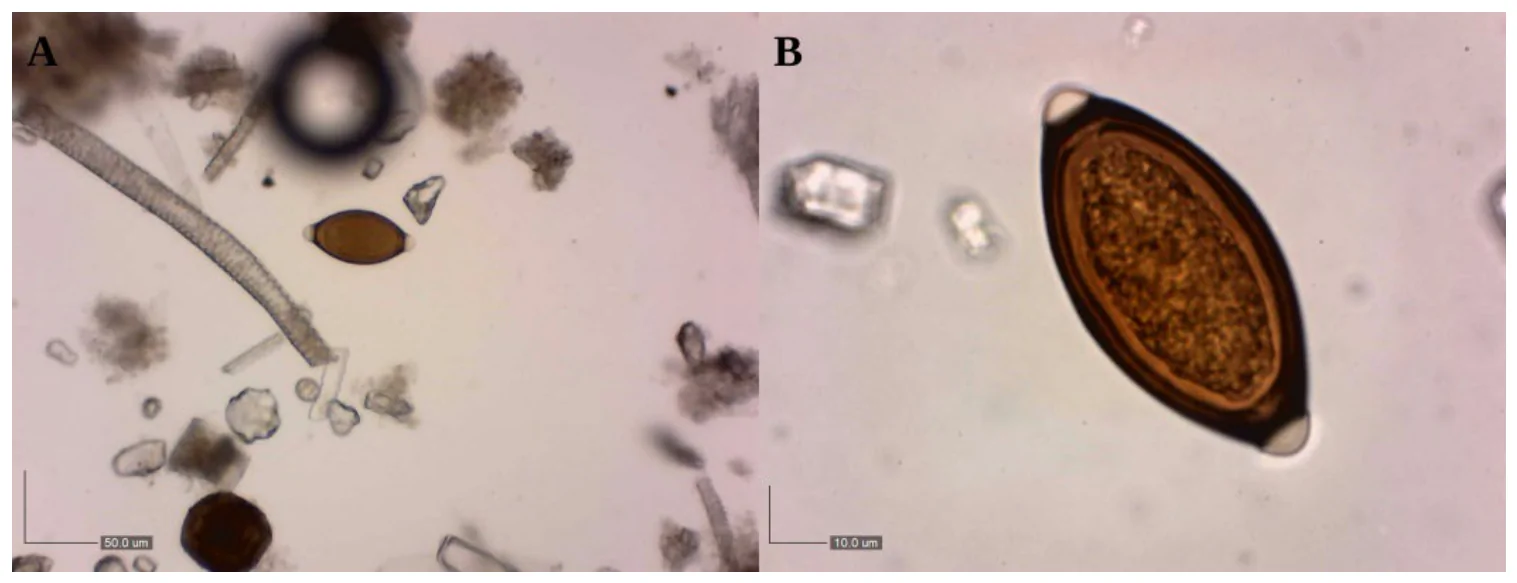
Eggs of *Trichuris* spp. from sheep feces, sedimentation method (light microscopy; (**A**) 100×; (**B**) 400×; original).

Coprological examination using concentration methods is the basis of routine diagnosis. The eggs are barrel- or lemon-shaped, with distinct polar plugs and a thick, layered, yellowish-brown shell, measure 50–80 × 30–50 µm, and are unembryonated when shed [12,28,37,79,102] (Figure 3). The morphology and dimensions of the eggs of different *Trichuris* species in ruminants overlap considerably, so a coprological finding confirms infection with *Trichuris* species but does not identify the species of the parasite [1,7,103]. To obtain reference egg material, Pasechnik patented a procedure for the in vitro collection of mature *T. ovis* eggs from adult females recovered from the colon of naturally infected animals [104].

Quantitative methods (McMaster, Mini-FLOTAC, FLOTAC) are used to estimate infection intensity and to monitor treatment efficacy, and they have also been applied in recent epidemiological studies in small ruminants [39,42]. Their sensitivity for *Trichuris* eggs at low egg counts, however, is limited. With the McMaster method, eggs of various helminth species, including *T. vulpis*, were reliably detected only from 500 eggs per gram of feces (EPG) upward, and with the combined sedimentation–flotation method with ZnSO_4_ (specific gravity [SG] 1.30), from 80 EPG upward [105]. In camels, the prevalence of *Trichuris* spp. was 0.3% with the Mini-FLOTAC method, 0.7% with the McMaster method, and 1.7% with semi-quantitative flotation [106].

According to the standard division of helminth eggs into light and heavy eggs, trichurid-type eggs are classified as light [107,108]. Nevertheless, several studies indicate that the choice of flotation solution and flotation time is particularly important for these eggs. In low-intensity infections, Alcaino and Baker [107] recovered *T. ovis* eggs by centrifugal flotation with sodium dichromate (SG 1.35) that had not been detected by flotation without centrifugation using sodium nitrate (SG 1.18–1.20); the ratio of the numbers of eggs recovered by the two methods was 2.4 for strongylid-type eggs, 3.2 for ascaridid eggs, and 6 for trichurid-type eggs. Flotation with sodium nitrate at a specific gravity of 1.30 recovered 62.7% more *Trichuris* eggs than the recommended specific gravity of 1.20 [109]. In sheep, flotation with NaCl detected 2.3 times fewer *T. ovis* eggs than a combined solution of Ca(NO_3_)_2_, sucrose, and NaCl [110], and in zoo ruminants, sugar solutions with a specific gravity of 1.27–1.40 were more efficient than MgSO_4_ (SG 1.26) and ZnSO_4_ (SG 1.18) solutions [111]. *Trichuris* eggs move more slowly in flotation solution than the eggs of other species, so at least 24 min was required to recover at least 80% of the eggs [112]. On the other hand, flotation with saturated NaCl solution detected *Trichuris* eggs in all lambs in the reported case of infection [7], which shows that this method is useful in infections of higher intensity.

These data are consistent with observations from the authors’ routine coprological diagnostic work, according to which methods intended for heavy helminth eggs (sedimentation, flotation with ZnSO_4_) and prolonged gravitational flotation are more sensitive for *T. ovis* eggs than flotation with saturated NaCl solution (SG 1.20). Whether *T. ovis* eggs differ in density from the eggs of other species of the genus has not been investigated; this hypothesis requires a quantitative comparison of methods using samples with known egg counts. Until then, flotation solutions of higher specific gravity (≥1.27–1.40), centrifugal flotation, or prolonged gravitational flotation are recommended for the diagnosis of *T. ovis*.

### 6.3. Eggshell Structure and Its Diagnostic Relevance

According to the classical trilaminar model, based on transmission electron microscopy of *T. suis* and *T. muris* eggs, the eggshell consists of an outer vitelline (lipoprotein) layer, a middle chitinous layer, and an inner lipid layer [113]. The chitinous layer has a lamellar organization, with chitin microfibrils enclosed in a protein coat, whereas the polar plugs contain less protein, which is arranged as fibrils within the chitin matrix, making the plugs susceptible to degradation during hatching [113]. In *T. trichiura*, more than 10 dense lamellae have been described in the chitinous part of the shell [114]. For *T. ovis*, only histochemical data are available, according to which proteins make up the major part of the internal contents of fully mature eggs [115].

On the basis of a critical analysis of the literature, Bond and Huffman [116] proposed replacing the trilaminar model with a hexalaminar framework adapted from the model developed for *Caenorhabditis elegans* [117]. In this framework, the outer layer (formerly the vitelline layer) is designated the *pellicula ovi* (layer 1) and is followed by the chitinous layer (layer 2), whereas the lipid-rich layer (layer 3a) is retained from the classical model; layer 5 represents the permeability barrier membrane, and layers 2 and 3 are each divided into two sublayers. The authors also extended the framework to the polar plugs of trichinelloid nematodes. We present the six components of this model as an ultrastructural framework for *Trichuris* eggs, bearing in mind that none of these layers has been experimentally characterized in *T. ovis*.

The thick, lamellar chitinous shell explains the high environmental resistance of the eggs and might also affect their density and, consequently, their behavior in flotation solutions. This relationship has not yet been investigated and represents a hypothesis that should be tested by measuring the density of the eggs of different *Trichuris* species. The thick chitinous shell also hinders DNA extraction from eggs, which limits the application of molecular methods to fecal samples.

### 6.4. Post-Mortem Diagnosis and Morphological Identification of Adults

At necropsy, adult worms are found embedded in the mucosa of the cecum and proximal colon, and the number of worms allows the intensity of infection to be estimated. Gross and histopathological changes are described in Section 5. Histopathological and immunohistochemical analyses are not part of routine diagnosis, but they are important research tools for studying the pathogenesis and the local immune response [7,79].

Morphological and morphometric analysis of adults is the standard method of species confirmation when molecular diagnosis is not available [7]. The worms are preserved in 70% ethanol, cleared in a mixture of glycerol and ethanol, and examined under a light microscope [79], and identification is based on the criteria described by Soulsby [1], and Kuchai et al. [78].

The body of the adult consists of a thin anterior part, which contains the esophagus surrounded by stichocytes, and a wider posterior part, which contains the intestine and the reproductive organs [24,79]. The cuticle is transversely striated, with a prominent longitudinal bacillary band. The oral cavity contains a spear-like stylet; according to some authors, the lips are poorly developed, whereas others do not describe them [37,79,102]. Females are longer than males. The posterior end of the male is spirally coiled and bears a single spicule with a retractable sheath covered with spines and with a bulbous expansion at its distal end covered with shorter spines. The vulva is located near the junction of the esophagus and intestine, is protruding, and is covered with spine-like papillae, and the uterus contains eggs arranged in a single row [12,23,24,28,37,102]. The main morphometric characteristics are presented in Table 1.

Spicule length is considered the main characteristic for differentiating males [12,23,37], but it overlaps between *T. ovis* and *T. globulosa* (Section 2). Vulval shape is not a reliable criterion either: in molecularly identified females, Vejl et al. [12] described four vulval morphotypes, one of which occurs in both *T. ovis* and *T. discolor*. Morphological identification should therefore be confirmed by molecular methods whenever possible.

### 6.5. Molecular Diagnosis

Because the morphological characteristics of the eggs and adults of *Trichuris* species in ruminants (*T. ovis*, *T. discolor*, *T. skrjabini*, *T. globulosa*) overlap, molecular methods are now the principal tool for reliable species identification, differential diagnosis, epidemiological and phylogenetic studies, and assessment of zoonotic potential [13,14,118]. The most important markers and their applications are presented in Table 2.

Ribosomal markers differ in their resolution. The partial 18S rRNA gene is highly conserved and suitable for confirming assignment to the genus, but not for distinguishing closely related species [14,118]. The ITS1 and ITS2 regions are more variable and reliably distinguish *T. ovis* from *T. discolor* [12,77], but ITS2 does not separate *T. ovis* and *T. globulosa*, for which mitochondrial markers are required [5,15]. No single marker meets all requirements, so a combined analysis of nuclear and mitochondrial markers is recommended [13,14,119].

Data on the genetic diversity of *T. ovis* depend on the marker used and the region studied. Low intraspecific variability (0–1%) based on the *cox1* gene was found in goats in Hunan Province [13], and similar results were obtained for *cox1*, *nad5*, and *cytb* in Guangdong Province [26]. In contrast, analysis of the ITS1-5.8S-ITS2 region in the Czech Republic revealed high haplotype diversity in *T. ovis*, together with genetically homogeneous *T. discolor* [12]. Hughes et al. [120] attributed low *cox1* haplotype diversity to a possible founder effect, but this conclusion rests on a limited sample from a single region.

The PCR markers INDEL1 and INDEL2, developed on the basis of stable insertions and deletions (INDELs) in the ITS region, allow *T. ovis* and *T. discolor* to be distinguished without sequencing, and their results were fully consistent with sequencing and morphological identification [12]. Using these markers, both species were detected in sheep and in roe deer, including mixed infections.

A particular problem is the analysis of DNA from eggs in feces, which is the only material available in routine diagnosis. In macaques, the partial 18S rRNA gene was amplified from 6 of 13 egg samples, whereas amplification of the ITS2 region was unsuccessful [14]. In addition, most of the available *T. ovis* sequences originate from China, several European countries, and Central Asia, whereas molecular data are lacking for many regions, including the Balkans [13,75].

Quantitative PCR (qPCR), next-generation sequencing (NGS), and metabarcoding represent a promising direction of development, as they provide higher sensitivity and allow mixed infections to be detected directly in fecal samples [121,122,123,124]. In humans, qPCR detected *Trichuris* eggs from as few as 5 EPG, compared with 50 EPG for microscopic methods [109], and the length of the ITS2 fragment, determined by nanopore sequencing, proved to be an inexpensive marker for distinguishing *T. trichiura* from *T. incognita* [9]. A similar approach could be developed to distinguish *Trichuris* species in ruminants, but such methods have not yet been developed or validated for *T. ovis* (Table 3).

## 7. Treatment and Control

### 7.1. Anthelmintics and Their Efficacy Against T. ovis

Treatment of trichuriosis should be regarded as part of an integrated control program that includes deworming, preventive measures, and herd management [124]. Four classes of anthelmintics are used in small ruminants: benzimidazoles (fenbendazole, oxfendazole, albendazole), macrocyclic lactones (ivermectin, moxidectin), imidazothiazoles (levamisole), and tetrahydropyrimidines (pyrantel, morantel) [125]. Methyridine, fenbendazole, and oxfendazole have been reported for the treatment of trichuriosis in ruminants [4]. Jalajakshi et al. [126] described the treatment of three goats with diarrhea, weakness, and chronic weight loss using oxfendazole and supportive therapy: two animals recovered within a week, whereas one died, and necropsy revealed a mixed infection with *Trichuris* and *Haemonchus* species.

Data on the efficacy of individual drugs specifically against *T. ovis* are very scarce, because studies of anthelmintic efficacy in small ruminants deal mainly with strongylids. In a study of naturally infected sheep in Ethiopia, *Trichuris* was among the nematodes that survived treatment with albendazole, ivermectin, levamisole, and tetramisole, whereas combination therapy resulted in a complete reduction in egg counts [127]. A systematic evaluation of anthelmintic efficacy against *T. ovis* therefore remains one of the priorities for future research.

Fenbendazole is relatively safe even at high doses, whereas albendazole should not be used during the first 30 days of pregnancy because of its embryotoxicity [126]. Because goats metabolize drugs faster, doses 1.5–2 times higher than those approved for sheep are often used in goats, although the effective dose for goats has not been established; underdosing may reduce efficacy and contribute to the development of resistance. The importance of metabolic differences is also indicated by the lower efficacy of ivermectin in goats treated at the dose for sheep [128].

### 7.2. Anthelmintic Resistance and Molecular Markers

Anthelmintic resistance is the genetically determined ability of parasites to survive treatment with a drug that was previously effective; after treatment, the resistant individuals survive and pass the resistance genes on to their offspring [129]. Resistance of nematodes of small ruminants to all major classes of anthelmintics is now widespread [129,130,131,132]. In India, resistance to fenbendazole and levamisole was found on all goat farms examined and resistance to ivermectin on 94.1% of them [133], and in Brazil, multidrug-resistant nematodes were found in all 15 sheep flocks examined [134]. These data, however, refer primarily to strongylids, above all *Haemonchus contortus*, whereas resistance in *T. ovis* has not yet been documented.

In nematodes of ruminants, benzimidazole resistance is associated with polymorphisms at codons 167, 198, and 200 of the isotype 1 *β-tubulin* gene. In *T. trichiura*, this approach has been investigated because of the moderate efficacy of albendazole: after treatment, the proportion of the homozygous resistant genotype at codon 200 increased from 3.1% to 55.3% in Haiti and from 51.3% to 67.8% in Kenya, with egg reduction rates of 49.7% and 10.1%, respectively [135]. Sequencing of the entire *β-tubulin* gene before and after albendazole treatment, however, showed no association between known polymorphisms and treatment response, suggesting that resistance markers in the genus *Trichuris* are probably also located outside this gene [136]. The *β-tubulin* gene has not been investigated in *T. ovis*, although assays for genotyping individual eggs from feces have been developed for *T. trichiura* [137] that could be adapted to this species.

### 7.3. Integrated Control

Current recommendations for the control of nematodes in small ruminants are based on integrated parasite management, which aims to reduce dependence on anthelmintics [16,138]. The key principles are as follows: avoiding treatment of all animals without prior coprological examination; preserving refugia, i.e., the part of the parasite population not exposed to the drug, through targeted selective treatment of animals based on fecal egg counts or body condition; monitoring drug efficacy with the fecal egg count reduction test (FECRT); and quarantining newly acquired animals, with coprological examination and treatment before they are introduced into the herd [16,138]. In practice, these principles are rarely applied: in southern Italy, only 24.4% of sheep farmers and 11.3% of goat farmers performed coprological examination before treatment [39], and smallholders are often unaware of correct dosing and deworming regimens [139].

The biology of *T. ovis* requires these measures to be adapted. Because embryonated eggs remain infective for years, pasture resting and rotation are less effective than for strongylids, whose larvae survive on pasture for a much shorter time. Reducing environmental contamination is therefore particularly important in trichuriosis: maintaining the hygiene of housing and outdoor pens, regular removal of feces, avoiding overcrowding, and feeding animals from feeders rather than from the ground [139,140]. Young animals, in which the most intense infections occur, should be given priority in control programs. Adequate nutrition with sufficient protein contributes to the resistance of animals to parasitic infections in general [141].

Among alternative methods, ovicidal fungi are of particular importance. The predatory fungus *Duddingtonia flagrans* acts on nematode larvae in feces and therefore has no direct effect on *Trichuris*, whose larva remains inside the egg. In contrast, *Pochonia chlamydosporia* penetrated and destroyed *T. vulpis* eggs in vitro, with a type 3 effect (hyphal penetration and colonization of the egg) of up to 94.8% after 21 days for one isolate, whereas *D. flagrans* and *Monacrosporium thaumasium* had no such effect [142]. *Mucor circinelloides* and *Trichoderma atrobrunneum* rendered 50% of *Trichuris* sp. eggs from camel feces nonviable within 30 days [143], and the administration of gelatin treats containing spores of *M. circinelloides* and *D. flagrans* to bison reduced the viability of *Trichuris* sp. eggs by 9–57% over four weeks [144]. Such formulations have not been tested in sheep and goats, but they represent a promising approach to reducing environmental contamination. Medicinal plants are also being investigated as complementary measures [16].

No vaccines against *Trichuris* species are available for ruminants. Proteins with WAP and CAP domains and other components of the ES products described in *T. muris* (Section 5) are being considered as potential candidates [99], but such studies have not yet been conducted for *T. ovis*.

## 8. Conclusions

*Trichuris ovis* is a widely distributed nematode of domestic ruminants whose importance should be assessed not only on the basis of clinical signs but also in the context of infection intensity, subclinical production losses, and the conditions that enable its persistence in herds. The direct life cycle and the long-term viability of eggs in the environment are the main factors in the maintenance and spread of infection. Prevalence data show marked geographic variability, but they should be interpreted with due regard to the diagnostic method used, because coprological findings do not allow *Trichuris* species to be distinguished. Molecular studies have confirmed the status of *T. ovis* as a species distinct from *T. discolor* and *T. skrjabini*, but they have also raised questions about its relationship with *T. globulosa* and the existence of cryptic genetic lineages. Infection control requires an integrated approach based on diagnosis, rational use of anthelmintics, and the preservation of refugia, with particular emphasis on reducing environmental contamination.

The reviewed literature also shows clear imbalances. Considerable attention has been given to prevalence surveys based on coprological examination, which cannot identify the species, and to the *T. muris* model of immunity, whereas studies combining morphological and molecular identification of *T. ovis* remain few, and the immune response of ruminants has been examined immunohistochemically in only a single flock. The relationship between *T. ovis* and *T. globulosa* and the existence of cryptic lineages remain controversial because ribosomal and mitochondrial markers give discordant results. Prevalence studies are most numerous from Europe and from South and Central Asia, whereas Africa, the Americas, and Oceania are represented by few studies; molecular data originate mainly from China, Spain, and the Czech Republic, and none are available from the Balkans. Priorities for future research therefore include integrative species identification in epidemiological surveys, evaluation of anthelmintic efficacy and resistance specifically in *T. ovis*, characterization of the immune response of ruminants, and the development of validated molecular diagnostic tools for fecal samples.

From a diagnostic perspective, coprological examination remains the basis of routine practice, but reliable species identification requires a combined analysis of nuclear (ITS1, ITS2) and mitochondrial markers, together with standardized protocols for DNA extraction from eggs in feces, whereas quantitative PCR and next-generation sequencing could enable the direct detection of mixed infections in fecal samples.

## Figures and Tables

**Table 1:** Morphometric characteristics of adult *Trichuris ovis*.

Parameter	Males	Females	References
Body length (mm)	29.54–85	32–87	[23,24,28,37,79]
Ratio of anterior to posterior body part	1.5–2:1	2.5–3:1	[24,28,37,102]
Width of anterior part (mm)	≈0.19 (0.175–0.220)	≈0.13 (0.11–0.18)	[12,24,37,102]
Width of posterior part (mm)	≈0.72 (0.63–0.76)	≈0.58 (0.23–0.83)	[12,24,37,102]
Spicule length (mm)	4.4–6.9	–	[23,24,28,37]
Vulva (length × width, mm)	–	0.08–0.16 × 0.05–0.16	[12]

**Table 2:** Molecular markers for the identification and characterization of *Trichuris ovis*.

Marker	Application	Limitations	References
18S rRNA (nuclear)	Genus assignment; phylogeny of more distantly related species	Low resolution for closely related species	[14,119]
ITS1, ITS2 (nuclear)	Differentiation of *T. ovis* and *T. discolor*; haplotypes; basis for insertion–deletion PCR (INDEL-PCR)	Does not separate *T. ovis* and *T. globulosa*; amplification from eggs unreliable	[5,12,14,15,77]
*cox1* (mitochondrial)	Species delimitation; haplotypes; molecular epidemiology	Data from few regions	[13,26]
*cob*/*cytb*, *nad5* (mitochondrial)	Delimitation of species and genetic lineages (*T. ovis*/*T. globulosa*)	Few sequences in databases	[5,26]
12S, 16S rRNA (mitochondrial)	Cryptic species and species complexes	Few sequences in databases	[3,15]
Complete mitochondrial genome	Species status; source of new markers	Requires larger amounts of high-quality DNA	[10,11]

**Table 3:** Diagnostic methods for *Trichuris ovis* infection: advantages, limitations, and applications.

Method	What it Detects	Advantages	Limitations	Application
Flotation with NaCl (SG 1.20)	*Trichuris* spp. eggs	Simple, inexpensive, rapid	Lower sensitivity in low-intensity infections; species not determined	Routine
Flotation with higher-density solutions (≥1.27–1.40), centrifugal or prolonged flotation	*Trichuris* spp. eggs	Higher egg recovery	More debris and faster crystallization of the solution	Routine, recommended
Sedimentation; combined sedimentation–flotation	*Trichuris* spp. eggs	Also detects heavier eggs	Longer examination of the sediment	Routine, complementary
Quantitative methods (McMaster, Mini-FLOTAC, FLOTAC)	Eggs per gram of feces (EPG)	Assessment of infection intensity and treatment efficacy	Low sensitivity at low egg counts	Routine, research
Necropsy and adult morphology	Species; worm burden	Species identification without expensive equipment	Post-mortem only; overlapping characteristics	Diagnostic, research
Histopathology, immunohistochemistry	Lesions; cellular immune response	Insight into pathogenesis	Post-mortem only; does not determine the species	Research
PCR and sequencing (18S, ITS, mitochondrial markers)	Species; haplotype; genetic lineage	Reliable identification and phylogeny	Cost; DNA extraction from eggs	Research, reference
INDEL-PCR (ITS)	*T. ovis* vs. *T. discolor*	No sequencing required	Validated on a small number of isolates	Research
qPCR, NGS, metabarcoding	Species; mixed infections; intensity	Highest sensitivity	Not yet developed or validated for *T. ovis*	Future development

## Data Availability

No new data were created or analyzed in this study. Data sharing is not applicable to this article.

## Abbreviations

The following abbreviations are used in this manuscript:

CAP	cysteine-rich secretory proteins, antigen 5, and pathogenesis-related 1 protein
CD	cluster of differentiation
*cob*/*cytb*	cytochrome b gene
*cox1*	cytochrome c oxidase subunit 1 gene
CXCL10	C-X-C motif chemokine ligand 10
DNA	deoxyribonucleic acid
EPG	eggs per gram of feces
ES	excretory–secretory
FECRT	fecal egg count reduction test
IFN-γ	interferon gamma
IL	interleukin
INDEL	insertion–deletion
ITS	internal transcribed spacer
L1	first-stage larva
mt	mitochondrial
*nad5*	NADH dehydrogenase subunit 5 gene
NGS	next-generation sequencing
NK	natural killer
PCR	polymerase chain reaction
qPCR	quantitative polymerase chain reaction
rRNA	ribosomal RNA
SG	specific gravity
Th	T helper
TNF-α	tumor necrosis factor alpha
*TPI*	triosephosphate isomerase
WAP	whey acidic protein
